# Integrating Phosphoproteomics and Bioinformatics to Study Brassinosteroid-Regulated Phosphorylation Dynamics in *Arabidopsis*

**DOI:** 10.1186/s12864-015-1753-4

**Published:** 2015-07-18

**Authors:** Li-Ling Lin, Chia-Lang Hsu, Chia-Wei Hu, Shiao-Yun Ko, Hsu-Liang Hsieh, Hsuan-Cheng Huang, Hsueh-Fen Juan

**Affiliations:** Department of Life Science, National Taiwan University, No. 1, Sec. 4, Roosevelt Road, Taipei, 106 Taiwan; Institute of Molecular and Cellular Biology, National Taiwan University, No. 1, Sec. 4, Roosevelt Road, Taipei, 106 Taiwan; Institute of Plant Biology, National Taiwan University, No. 1, Sec. 4, Roosevelt Road, Taipei, 106 Taiwan; Institute of Biomedical Informatics, Center for Systems and Synthetic Biology, National Yang-Ming University, No.155, Sec.2, Linong Street, Taipei, 112 Taiwan; Graduate Institute of Biomedical Electronic and Bioinformatics, National Taiwan University, No. 1, Sec. 4, Roosevelt Road, Taipei, 106 Taiwan

**Keywords:** Phosphoproteomics, Bioinformatics, Brassinosteroids, Phosphorylation dynamics, Kinase-centered network

## Abstract

**Background:**

Protein phosphorylation regulated by plant hormone is involved in the coordination of fundamental plant development. Brassinosteroids (BRs), a group of phytohormones, regulated phosphorylation dynamics remains to be delineated in plants. In this study, we performed a mass spectrometry (MS)-based phosphoproteomics to conduct a global and dynamic phosphoproteome profiling across five time points of BR treatment in the period between 5 min and 12 h. MS coupling with phosphopeptide enrichment techniques has become the powerful tool for profiling protein phosphorylation. However, MS-based methods tend to have data consistency and coverage issues. To address these issues, bioinformatics approaches were used to complement the non-detected proteins and recover the dynamics of phosphorylation events.

**Results:**

A total of 1104 unique phosphorylated peptides from 739 unique phosphoproteins were identified. The time-dependent gene ontology (GO) analysis shows the transition of biological processes from signaling transduction to morphogenesis and stress response. The protein-protein interaction analysis found that most of identified phosphoproteins have strongly connections with known BR signaling components. The analysis by using Motif-X was performed to identify 15 enriched motifs, 11 of which correspond to 6 known kinase families. To uncover the dynamic activities of kinases, the enriched motifs were combined with phosphorylation profiles and revealed that the substrates of casein kinase 2 and mitogen-activated protein kinase were significantly phosphorylated and dephosphorylated at initial time of BR treatment, respectively. The time-dependent kinase-substrate interaction networks were constructed and showed many substrates are the downstream of other signals, such as auxin and ABA signaling. While comparing BR responsive phosphoproteome and gene expression data, we found most of phosphorylation changes were not led by gene expression changes. Our results suggested many downstream proteins of BR signaling are induced by phosphorylation via various kinases, not through transcriptional regulation.

**Conclusions:**

Through a large-scale dynamic profile of phosphoproteome coupled with bioinformatics, a complicated kinase-centered network related to BR-regulated growth was deciphered. The phosphoproteins and phosphosites identified in our study provide a useful dataset for revealing signaling networks of BR regulation, and also expanded our knowledge of protein phosphorylation modification in plants as well as further deal to solve the plant growth problems.

**Electronic supplementary material:**

The online version of this article (doi:10.1186/s12864-015-1753-4) contains supplementary material, which is available to authorized users.

## Background

Brassinosteroids (BRs) are steroid plant hormones essential for normal plant growth and development. Numerous plant processes are regulated by BRs, including cell elongation and division, photosynthesis, photomorphogenesis, flowering, seed germination, root development and abiotic/biotic stress response [1–5]. Therefore, understanding the response mechanism of BR regulation is critical for promoting agricultural development and boosting crop productivity by improving plant growth conditions.

Extensive studies using genetic and molecular approaches have identified major BR signaling components, which indicate that protein phosphorylation plays a crucial role in BR regulations [3, 6, 7]. The cell surface receptor complex containing the receptor-like kinase, BR-insensitive 1 (BRI1), responds to BR signaling components by inducing the initial phosphorylation-dependent signal transduction cascade [3]. The binding of BR to the extracellular domain of BRI1 leads to auto-phosphorylation of BRI1 which phosphorylate BRI1 KINASE INHIBITOR 1 (BKI1), and then induces trans-phosphorylation between BRI1 and BRI1-associated receptor kinase1 (BAK1). Activated BRI1 phosphorylates BR-signaling kinases (BSKs) and BR-insensitive 2 (BIN2) which can transduce its signals to BRI1 suppressor1 (BSU1) phosphatase [6], and then stops phosphorylation of Brassinazole-resistant1 (BZR1)/BRI1-ems-suppressor 1 (BES1) to induce BR-responsive target gene expression [8]. In negatively regulated stomatal cell formation, the activation of BIN2 could be reduced by BR, to inhibit mitogen-activated protein kinase kinase 4, 5, 7 and 9 (MKK4/5/7/9), which induce the phosphorylation of mitogen-activated protein kinase 3 and 6 (MPK3/6) [9]. In the immune response, BAK1 phosphorylation interacts as a co-receptor with BRI1-induced BR signaling and flagellin-sensing 2 (FLS2) signaling to increase disease resistance [10]. In brief, BSKs activate downstream phosphorylation signals to result in expression of BR target genes and to regulate stomatal formation, innate immunity, and stress responses during plant development.

Several proteomics studies also identified many proteins which are post-translationally modified in response to BR [11–14]. Huang and colleagues used two-dimensional (2-D) gel electrophoresis to investigate the regulatory relationship between BR and chilling condition in mug been epicotyl and found BR-induced proteins are involved in methionine assimilation, ATP synthesis, cell wall construction and the stress response [11]. Deng and colleagues identified posttranslational modification of BiP2, a luminal binding protein under BR treatment using 2-D difference gel electrophoresis (DIGE) [12]. Similarly, another study combining prefractionation with 2-D DIGE in *Arabidopsis* identified phosphorylation sites of BZR1, two tetratricopeptide repeat proteins, a phosphoenolpyruvate carboxykinase (PCK1), and a novel BR-induced plasma membrane protein (DREPP) which are associated with BR promotion of plant growth [13]. Shigeta and colleagues identified several BR-responsive proteins located in the nucleus, including nucleosome assembly protein 1;1 (NAP1;1), NAP1;2, band 7 family protein, vernalization independence 3, s-adenosylmethionine synthetase 2, and 60S ribosomal protein L14, and found that NAP1;2 has a posttranslational modification in response to cellular BR levels [14]. Consequently, a comprehensive identification of phosphorylation events under the stimulus of BR is necessary to uncover the BR-regulated signaling networks.

Recent advances in phosphoproteomics, including high-accuracy mass spectrometry (MS) and phosphopeptide-enrichment techniques, have allowed identification of previously nearly undetectable site-specific phosphorylations in plants [15]. Current methods for enriching phosphopeptides prominently include strong cation exchange (SCX) chromatography, immobilized metal-affinity chromatography (IMAC), and titanium dioxide (TiO_2_) chromatography. SCX separates phosphopeptides from non-phosphopeptides by their solution charge state, but is unsuitable as a stand-alone method [16]. IMAC and TiO_2_ affinity chromatography are commonly used for phosphoproteome enrichment because of their high affinity for phosphopeptides. However, conventional IMAC suffers from partial non-specific absorption resulting from acidic non-phosphopeptides, though it can be improved by methyl esterification [17, 18]. Recently, the method of aliphatic hydroxy acid-modified metal oxide chromatography (HAMMOC) was invented to substantially improve the efficiency and specificity of TiO_2_ phosphopeptides enrichment [19]. It can reduce the interaction between non-phosphopeptides and metal oxides, thus removing most non-phosphopeptides from samples.

Although several studies have profiled the protein expression under BR response by MS techniques [11–14, 20–22], the dynamics of protein phosphorylation in response to BR induction is not comprehensively identified till now. To capture the dynamics of phosphorylation events under BR regulatory responses, we performed a time-dependent study in *Arabidopsis* cell suspension cultures with 24-epibrassinolide (a highly active BR). Dimethyl labeling and HAMMOC combined with nano-liquid-chromatography-tandem mass spectrometry (nanoLC-MS/MS) was used to characterize the phosphoproteome of BR signaling processes.

However, because MS-based proteomics tends to have data consistency (poor reproducibility and inter-sample agreement) [23] and coverage (inability to detect the entire proteome) [24] issues, it is difficult to profile all phosphorylation sites at whole time points. Several technical approaches, such as exhaustive fractionation of samples [25] and repeated MS runs of the same samples [26], are widely used to address these issues, but these are still unable to completely overcome the problems. Recent studies have used bioinformatics approaches, such as biological networks [27], to complement the existing experimental approaches to increase the comprehensiveness of proteome coverage and enhance analytical resolution. Therefore, we used several bioinformatics approaches, including enrichment analysis as well as construction of protein-protein and kinase-substrate interaction networks to reveal the phosphorylation dynamics of BR in *Arabidopsis*.

## Results

### To determine appropriate BR treatment concentrations in *Arabidopsis* cells

**Fig. 1:**
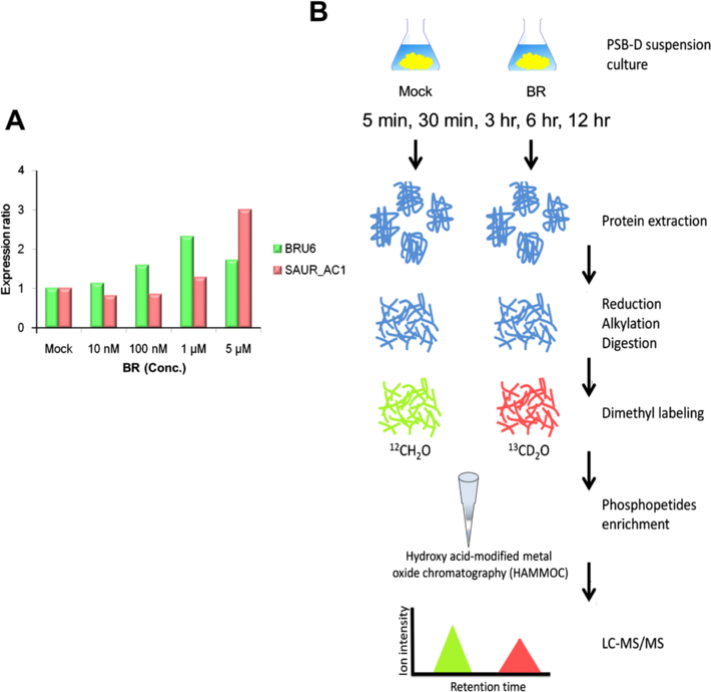
Phosphoproteomic study strategy. **a** To determine appropriate treatment concentrations, two BR-regulated genes of the expression level (*BRU6* and *SAUR-AC1*) were detected in different BR concentrations using qRT-PCR. The expression ratio shows gene expression of different BR concentrations against mock-treated RNA. The indicated concentrations were used for a PSB-D cell suspension culture for 3 h. 18S ribosomal RNA was assigned to the reference gene. **b** For phosphoproteomic study, PSB-D cells were treated with BR for 5 min, 30 min, 3 h, 6 h, and 12 h. Peptide dimethylation and titanium dioxide phosphopeptide enrichment were combined with nano-liquid chromatography–tandem mass spectrometry (nanoLC–MS/MS) analysis to characterize the phosphoproteome of the BR signaling process

Selection of suitable material for phosphoproteomic analysis was important for the investigation of the molecular mechanism of BR. PSB-D cells were chosen because of their short culturing time and growth condition characteristics, similar to those of root cells. To determine the most suitable concentration of exogenous BR24-epibrassinolide for cell treatment, we evaluated the activation of BR signaling in *Arabidopsis* cells based on the expression levels of two genes, small auxin up RNA (*SAUR-AC1*) [28] and indole-3-acetic acid amido synthetase (*BRU6*) [29], known to be upregulated by BR. Because previous studies have shown that the expression of *SAUR-AC1* and *BRU6* in five-day-old *Arabidopsis* cells were apparently changed after the treatment of BR for 3 h [30]. We found that *SAUR-AC1* and *BRU6* were clearly upregulated at concentrations of 1 and 5 μM (Fig. 1a), whereas higher expression of *SAUR-AC1* compared with *BRU6* was observed at 5 μM of 24-epibrassinolide which is also the same as used in previous *Arabidopsis* cells studies [31, 32]. Therefore, we used 5 μM of 24-epibrassinolide for the subsequent experiments.

### Characterization of the BR-regulated phosphoproteome in *Arabidopsis* cells

**Fig. 2:**
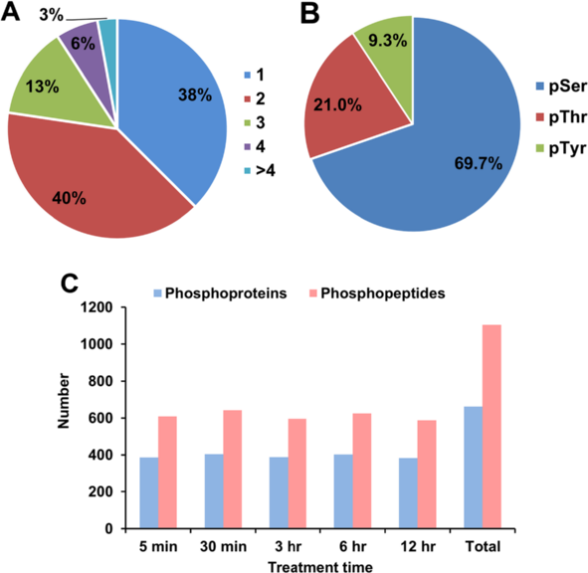
Characteristics of phosphorylated residues, phosphopeptides, and phosphoproteins data obtained in the study. **a** Frequency of phosphorylated residues distributed in the phosphopeptides: one phosphorylation site is 38 %, two phosphorylation sites is 40 %, three phosphorylation sites is 13 %, four phosphorylation sites is 6 % and more than four phosphorylation sites is 3 %. **b** Distribution of phosphorylation sites (class I) in the phosphoproteomic data. **c** Number of unique phosphopeptides and phosphoproteins identified in each time point experiment

To accurately determine the BR-regulated phosphoproteins in *Arabidopsis*, we performed a time-course phosphorylation experiment and applied a quantitative phosphoproteomic analysis approach (Fig. 1b). We collected samples from cells treated with 5 μM BR or mock at five time points: 5 min, 30 min, 3 h, 6 h, and 12 h. A total of 1104 unique phosphopeptides were identified, derived from 739 distinct phosphoproteins at a peptide FDR of < 1 %. Most of the identified phosphopeptides contained either one or two phosphorylation sites (Fig. 2a). Among all identified phosphorylation sites (Additional file 1), 1231 were considered as high-confidence hits (class I, phosphorylation localization probability > 0.75). Additionally, 249 phosphorylation sites were classified as class II (0.5 < *p* ≤ 0.75) and 158 as class III (*p* ≤ 0.5). In further analysis, only class I sites among 644 proteins were used. A total of 858 serines (Ser), 259 threonines (Thr), and 114 tyrosines (Tyr) were identified as high-confidence phosphorylation sites, yielding a Ser/Thr/Tyr phosphorylation ratio of 69.7/21.0/9.3 % (Fig. 2b). The number of identified phosphopeptides/phosphoproteins was similar among different time points (Fig. 2c and Table 1).

**Table 1:** The number of phosphorylation sites in the time-course analysis

	5 min	30 min	3 h	6 h	12 h
pS	550 (78.4 %)	604 (79.0 %)	564 (77.8 %)	592 (79.8 %)	560 (80.8 %)
pT	116 (16.5 %)	120 (15.7 %)	116 (16.0 %)	108 (14.6 %)	101 (14.6 %)
pY	36 (5.1 %)	41 (5.4 %)	45 (6.2 %)	42 (5.7 %)	32 (4.6 %)
total	702	765	724	742	693

Since MS-based proteomics have consistency and coverage issues, 693 out of 1231 high-confident phosphorylation sites were assigned a ratios at least one time point, but only 394 out of 1231 phosphorylation sites were quantified at all five time points (Additional file 2). Although these phosphorylation sites contained missing values, they might be still informative for understanding the dynamics of BR signaling via bioinformatics approaches. Therefore, a total of 1087 quantitative phosphorylation sites were considered for further analysis.

### Biological processes of *Arabidopsis* cells mediated by BR dynamic phosphosignaling

**Fig. 3:**
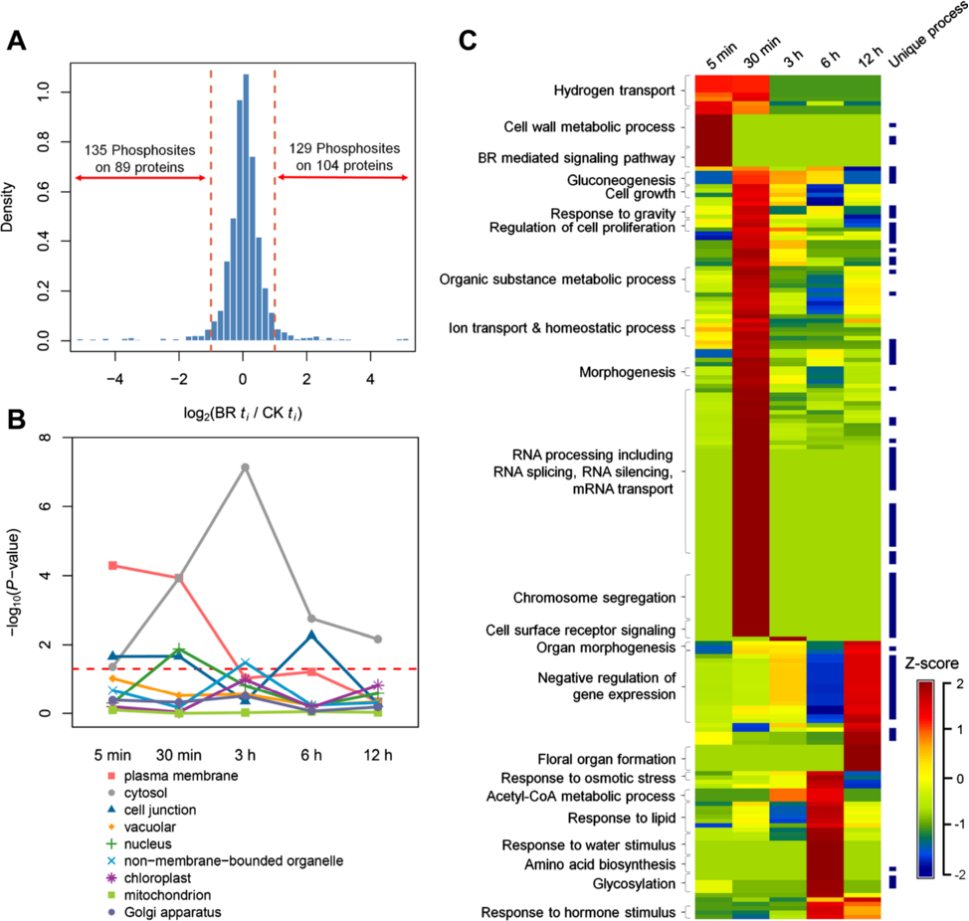
Functional groups in significantly regulated phosphoproteins. **a** Distribution of all quantified phosphopeptides in BR treatment and BR-responsive sites which has two-fold phosphorylation change. **b** The proteins with BR-responsive sites are annotated according to cellular compartment GO terms. The *P*-value is obtained from Fisher’s exact test with Benjamini-Hochberg correction. The *red dash line* denotes the *P*-value = 0.05. **c** The heatmap presents the dynamic change of biological process with similar analysis as in (b). The values in the heatmap represent the z-transformation of –log_10_ (*P*-value) by GO term (*Color scales*: *red*, high overrepresentation; *blue*, high underrepresentation). The unique GO terms are lighted in *dark blue*

To obtain functional insights into the biological processes and cellular organization dynamically affected by BR treatment, Gene Ontology (GO) enrichment analysis was conducted. A threshold of 2-fold phosphorylation change (e.g. |log_2_(fold-change)| ≧ 1) was applied to reveal 129 positively and 135 negatively BR-induced phosphorylation sites on 171 distinct proteins (Fig. 3a), and GO enrichment analysis was performed on these proteins. The proteins regulated at initial time points (BR treatment for 5 min) significantly localized on plasma membrane (Fig. 3b) and enriched on the biological process term “BR mediated signaling pathway” (Fig. 3c and Additional file 3). Indeed, BR signaling is initiated from a series of transmembrane receptors and receptor-like kinases which are located in plasma membrane [7]. With the increasing time of BR treatment, we observed the propagation of BR effects. In the annotation of cellular component ontology, phosphorylated proteins enriched from 5 min samples are mostly plasma membrane proteins, and enriched from 30 min samples are mostly cytosol and nucleus proteins (Fig. 3b). Interestingly, the phosphorylated proteins after 3 h were still enriched on cytosol compared with 6 and 12 h treatment data. We speculated that these proteins may be the BR-responsive genes in transcriptional level and activated or suppressed by phosphorylation. For example, acyl-CoA binding protein 4 (ACBP4), tubulin alpha-1 chain (TUA1) and tubulin alpha-4 chain (TUA4) located in cytosol are BR-responsive genes [6] and have also significantly phosphorylation change at 3 h. The similar trend was also observed in the annotation of biological process ontology (Fig. 3c). In addition to the biological process term “BR mediated signaling pathway”, phosphorylated proteins at 5 min also involved in “proton transport” and “cellulose metabolic process” which are likely to contribute to BR promotion of solute uptake and directional cell elongation [2]. At 30 min, phosphorylated proteins could be contributed to diverse processes, such as cell growth and proliferation, morphogenesis, and RNA processing (Fig. 3c). This might indicate that the signal delivers to nucleus for transcription after less than 30 min of BR treatment. These results of functional analyses from temporal phosphoproteomic experimental display the dynamic of BR signaling.

To clarify that these enriched biological processes are uniquely involved by BR-induced phosphoproteins, we compared the enriched GO terms using the BR-responsive phosphoproteins and genes derived from genome-wide transcriptional experiments [6, 33, 34]. A total of 4326 genes obtained from Wang and colleagues [6] show altered expression levels by BR treatment or by both *bri1* and *bzr1* mutations. After GO enrichment analysis, 532 GO biological process terms were enriched in the BR-responsive genes (adjusted *P*-value < 0.05). After comparing two sets of enriched GO terms, 98 GO terms were uniquely enriched on BR-induced phosphorylated proteins (Fig. 3c and Additional file 3). These unique processes include “gluconeogenesis”, “response to gravity”, “RNA silencing”, “RNA transport”, “chromosome segregation”, and “protein glycosylation”. These results suggest that these processes are regulated through proteins whose phosphorylation sites are induced by BR.

### Protein-protein interaction network unveils the possible BR-regulated network

**Fig. 4:**
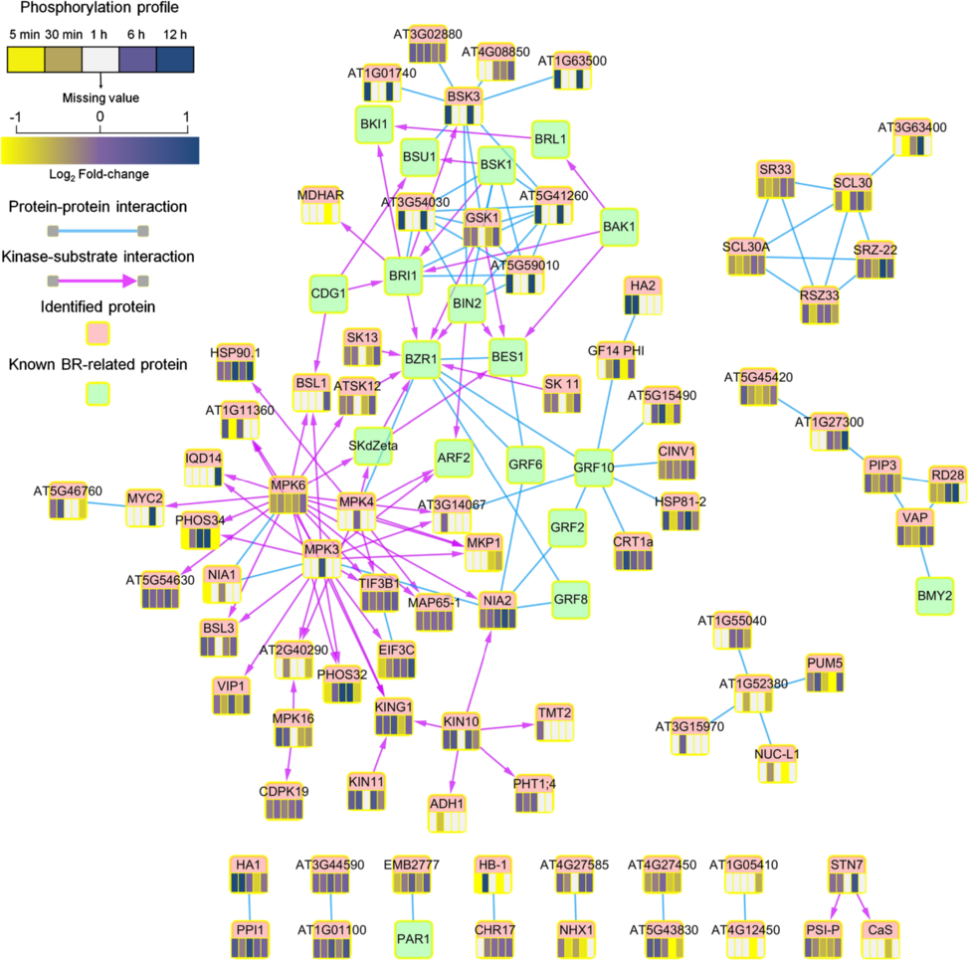
Protein-protein interaction network among identified and BR-related proteins. The identified and known BR-related proteins are presented by nodes and colored by *pink* and *green*, respectively. The *blue edge* and *purple arrow* indicate the experimental validated protein-protein and kinase-substrate interactions, respectively. The *color scales* represent the phosphorylation ratio at each time point. If the ratio is unavailable at a time point, the fill is colored by *white*

To better understand the relationships among all identified phosphoproteins, we constructed a protein-protein interaction network (PIN) derived from BioGRID [35], AtPID [36], and PhosPhAt [37]. Additionally, PhosPhAt also provides the known kinase-substrate interactions. The constructed PIN was composed of 99 proteins, including 18 BR-related proteins collected from literature [3, 6, 7] and 81 phosphoproteins identified in this study, and 144 interactions (Fig. 4). From the network, we found that many phosphoproteins identified in this study were known or strongly associated with core components of BR signaling. Several known BR signaling components were also identified in our experiment, including brassinosteroid-signaling kinase 3 (BSK3) [6], GSK3/Shaggy-like protein kinase 1 (GSK1) [38], MPK3/MPK6 [9, 39, 40], 14-3-3-like protein G-box factor 14 phi (GF14 PHI) [41], and BSU 1-like 1 and 3 (BSL1 and BSL3) [38].

In addition to these known BR signaling components, many phosphoproteins might be the BR signaling associated proteins according to the PIN results (Fig. 4). Besides BSK3, a group of receptor-like cytoplasmic kinases (RLCK) II family with the consensus phosphosites to BSK3, including AT5G59010, AT3G54030, and AT5G41260, interacts with upstream components of BR signals such as BRI1, BSK1, BSK3 and BIN2. These proteins were significantly up-regulated at 5 min, indicating that they may play the roles as co-regulated kinases in the initial steps of BR signaling. SHAGGY-related kinase 11 (SK11), SHAGGY-related protein kinase 12 (AtSK12), and SHAGGY-like kinase 13 (SK13) belonging to subgroup I of *Arabidopsis* GSK3/SHAGGY-like kinases are important in BR signaling by phosphorylating BZR1 [38]. Moreover, many proteins are the substrates of mitogen-activated protein kinase (MPK3, MPK4 and MPK6), and regulated at the late time points; it indicates that BR signalling could control divergent processes through the mitogen activated protein kinase (MAPK) pathway [9].

### Various kinases involved in the BR-transduced downstream phosphosignals

The sequence consensus of phosphopeptide motifs reflects the kinase-specific regulation of substrates and the identification of the corresponding kinases. To find primary initial protein phosphorylation inducers, we submitted the flanking sequences of high-confident phosphorylation residues to Motif-X. After motif analysis, 13 serine and 2 threonine significantly enriched motifs were identified, but no tyrosine motif was significantly enriched (Table 2). Through literature and database survey [37, 42, 43], some motifs corresponding kinases are well-known: pSDDE, pS[D/E]X[D/E] and p[S/T]XX[D/E] for casein kinase II (CK2) family; p[S/T]P for GSK-3, cyclin dependent kinase (CDK) and MAPK families; RXXpS for SNF1-related kinase II (SnRK2) family; pSXXS for casein kinase I (CK1) family. Although no kinases in plant can be assigned to DpS, EpS, and pSXP, they are similar to the recognized motifs of some kinases in human (Additional file 4): DpS for large tumor suppressor kinase 1 (LAST1) and NIMA-related kinase 2 (NEK2), EpS for pre-mRNA processing factor 4B (PRPF4B), and pSXP for casein kinase 1, gamma 3 (CSNK1G3), dual-specificity tyrosine-phosphorylation regulated kinase 4 (DYRK4), and PDLIM1 interacting kinase 1 like (PDIK1L). The motif, pSXDXE, has not yet been assigned to any specific kinases, it may be novel and interesting for further investigation.

**Table 2:** The enriched motifs of all identified phosphopeptides

Motif	Corresponding kinase	No. of occurrence	No. phosphorylation change (two-fold)^a^
pSDDE	CK2	32	5
pSDXE	CK2	99	16
pSDXD	CK2	73	5
pSP	GSK3, MAPK, CDK	139	24
pSEXE	CK2	58	7
pSXDXE	Unknown	41	3
DpS	Similar to human kinases: LAST1 and NEK2	141	20
EpS	Similar to human kinase: PRPF4B	133	23
RXXpS	SnRK2, CaMK	75	18
pSXP	Similar to human kinases: CSNK1G3, DYRK4, and PDIK1L	76	14
pSXXD	CK2	147	14
pSXXE	CK2	233	28
pSXXS	CK1	107	21
pTP	MAPK, CDK	34	9
pTXXE	CK2	36	7

^a^No. of occurrence on sites with two-fold phosphorylation change at any time point

**Fig. 5:**
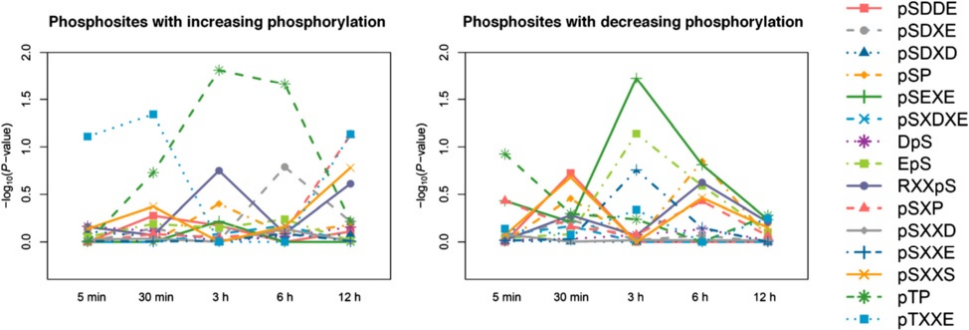
The time-dependent phosphorylation trend of substrates of kinase motifs. The *P*-value is obtained by Fisher’s exact test and used to assess whether phosphosites with increasing (*left* figure) and decreasing phosphorylation (*right* figure) under BR treatment compared to control are enriched on a certain motif of a given time point

Sequentially, we combined motifs with phosphorylation profiles to reveal the dynamics of BR signaling transduction (Fig. 5). Although only few motifs were considered as significant enrichment, i.e. *-*log_10_ (*P*-value) ≧ 1.3, at some time points, these analyses are useful to observe the activation profile of a specific kinase after BR treatment. The phosphorylation levels of proteins containing one of MAPK recognizing motifs pTP tend to be down-regulated in the initial steps and then up-regulated at the later time points. This phosphorylation trend is also observed in PIN where most of the substrates of MPK3/MPK6 were up-regulated at 6 or 12 h (Fig. 4), and consistent with the previous study in which MAPK may initially be suppressed by BR-regulated GSK3/Shaggy-like kinases [9]. Moreover, the phosphosites with motif pTXXE was increasingly phosphorylated at 5 min and 30 min, but those with motif pSDDE and pSEXE/pSXXE were decreasingly phosphorylated at 30 min and 3 h, respectively. The result suggests their corresponding kinase, CK2, plays a crucial role in BR signaling. Indeed, CK2 is important for cell growth and cell viability, and may make crosstalk between BR and other signaling, such as auxin and abscisic acid (ABA) signalling [44, 45]. In addition, SnRK2, a central component of ABA signaling, of which substrates (RXXpS) were increasingly phosphorylated at 3 h, suggesting the interaction between BR and ABA signaling pathways [46]. Among these motifs, the phosphosites with motif pSXP were decreasingly phosphorylated at 30 min. Although the corresponding kinase of motif pSXP in *Arabidopsis* is still unknown, previous study has also observed that this motif is enriched in the up-regulated phosphopeptides under osmotic stress [47].

### A dynamic phosphorylation network in BR-regulated *Arabidopsis* cells

**Fig. 6:**
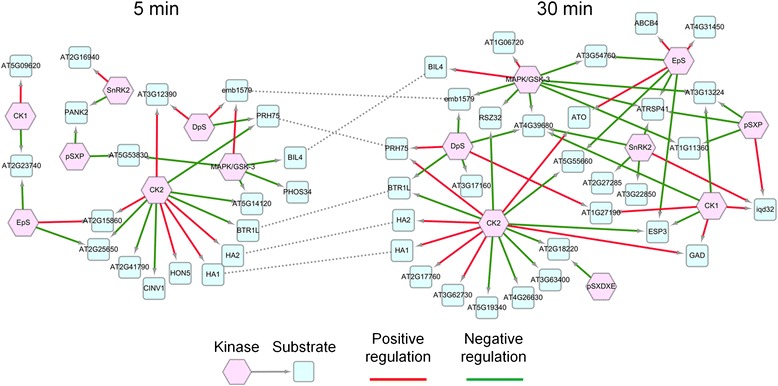
Time transition of kinase-substrate network from time point 5 min to 30 min. The kinase-substrate networks were constructed based on the enriched motifs of phosphoproteins. The *red hexagon* and *blue rectangle* nodes represent kinase and substrate, respectively. The *red* and *green arrows* represent the positive and negative phosphorylation regulation at the given time point. The node connected by *dash line* is the common protein appeared at both time points

On the basis of the results presented above, the relation between kinases and substrates were determined by identification of the enriched motifs at phosphorylation sites differential with more than two-fold. We focused on the transition of phosphorylation events from 5 min to 30 min and constructed time-dependent kinase-substrate networks for 5 and 30 min, respectively (Fig. 6). Several proteins have been demonstrated associated with the response of BR signaling. The phosphorylation of H(+)-ATPases (HAs), including HA1 and HA2, were persistently activated by CK2 during 5 and 30 min treatment. Indeed, the activation of H(+)-ATPase is necessary for the maintenance of homeostasis due to BR inductions of hyperpolarization and cell wall expansion [48]. Similarly, ATP-binding cassette sub-family B member 4 (ABCB4) was positively phosphorylated at 30 min treatment and may assist auxin transport for BR response [33]. This might also explain the persistent activation of auxin-responsive GH3-like protein (AT1G48660) after BR treatment (Additional file 1). Another interesting result, the phosphorylation of BZR-insensitive-long hypocotyls 4 (BIL4) was down-regulated at 5 min treatment and then up-regulated at 30 min by MAPK. This observation is consistent with the previous study that BIL4 acts as a slow effective factor for mediating cell elongation in BR signalling [49]. Leucine-rich repeat protein kinase family protein (AT1G27190), RING/U-box superfamily protein (AT4G31450), and hypothetical protein (AT5G19340) were significantly regulated at 30 min treatment by different kinases. Although the function of these proteins is still unclear, all of them reveal altered expression levels after BR treatment [33, 34]. Additionally, AT5G19340 is the BZR1 directly regulated gene [33]. IQ-domain 32 (Iqd32), a proteins containing IQ-domain, was up-regulated at 30 min treatment. It could facilitate cellular RNA localization as one mechanism to control and fine-tune gene expression and protein sorting for certain types of cellular signalling [50]. In the kinase-substrate network at 30 min treatment, several proteins have been reported that can be phosphorylated by ABA regulation, such as arginine/serine-rich-splicing factor 41 (ATRSP41), binding to Tomato mosaic virus RNA 1 Long form (BTR1L), serine/arginine-rich splicing factor Z32 (RSZ32; AT3G53500), iqd32, SAP domain-containing protein (AT4G39680), DEK domain-containing chromatin associated protein (AT4G26630), and adenine nucleotide alpha hydrolases-like protein (AT1G11360) [51]. To sum up, this constructed dynamic phosphorylation network can provide the possible downstream events of BR-induced signaling.

### Comparison of BR-induced phosphorylated proteins and expressed genes

**Fig. 7:**
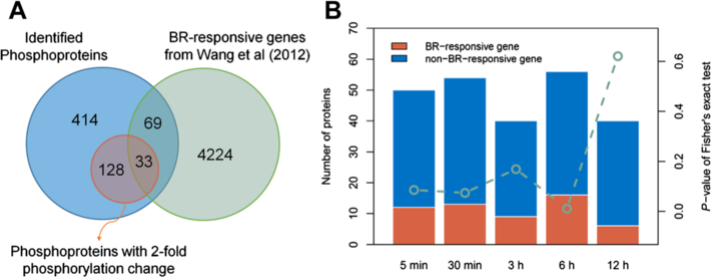
Comparison between phosphorylated proteins and BR-responsive genes in transcription level. **a** The Venn diagram shows the overlap between phosphorylated proteins and BR-responsive genes. **b** The proportion of significantly regulated phosphoproteins which are also BR-responsive genes (*red*) and not BR-responsive genes (*blue*) at each time point is presented by *bar plot*. The *green circle* indicates the significance of the proportion of BR-responsive genes which is assessed by Fisher’s exact test

Brassinosteroid induces not only phosphorylation change for signaling transduction, but also expression change for responding comprehensively biological processes. Therefore, the identified phosphorylation change may have a bias due to the expression change on transcriptional levels. To clarify this concern, we compared the identified phosphoproteins with the 4326 BR-responsive genes as previously described. 102 out of 644 identified phosphoproteins are also BR-responsive genes, including 55 BR-induced genes, 46 BR-repressed genes, and one gene that show complex expression in different experiment (Additional file 5). However, these two data were not significantly overlapped (*P-*value = 0.50, Fisher’s exact test) (Fig. 7a). If only considering the proteins with significantly phosphorylation changes, there were 33 proteins and the overlap is also not statistically significant (*P-*value = 0.066, Fisher’s exact test). In these 33 common proteins, the gene expression and phosphorylation regulation trend in most of them are consistent, but six proteins are not (Additional file 5). Furthermore, we examined whether the proteins with significant phosphorylation changes were enriched in BR-responsive gene for each time point. We found only significantly regulated proteins at 6 h were enriched in BR-responsive genes (*P*-value = 0.011, Fisher’s exact test) (Fig. 7b). These results reveal that the phosphorylation change measured in this study does not correlate with transcription change in RNA level. Additionally, these also suggest that BR-signaling not only induces gene expression which regulated by BZR1 and BES1, but also change protein activation by phosphorylation via various kinases.

## Discussion

BR stimulates the signaling by binding to transmembrane receptor complex to influence the activity of transcription factors, which regulate the expression of BR-responsive genes for a wild range of physiological processes. In addition to the influence of gene expression, recent studies indicated that some of the components of the BR signaling pathway involve in other signaling network to regulate diverse physiological processes [6, 7, 52]. In this study, we conducted a proteome-wide analysis of temporal phosphorylation to reveal the components of BR signaling and cross-talking pathways. A total of 1231 high confident phosphorylation sites belonging to 644 proteins were identified in three biological replicates. Of these, 239 phosphorylation sites were significantly influenced by BR that is in higher percentage than previous proteomic studies [13]. In this study, we profile the phosphorylation events responded to BR by the HAMMOC enrichment method coupling with mass spectrometry (nano LC-MS/MS) which provides high efficiency and specificity to detect phosphopeptides [19]. It might lead to more significantly different phosphorylation sites identified in this study. In the phosphorylated residue analysis, the proportion of phosphorylation on tyrosine residues (9 % ) was greater than in previous studies [15, 28, 29], but there were no differences between our time point experiments (Fig. 2 and Table 1).

Among these identified phosphoproteins, the key components of BR signaling, including BRI1, BAK1, BKI1, BSK1, BSU1, BZR1, and BES1, were not observed in this study. We examined five recently large-scale protein phosphorylation analyses [51, 53–56], and surprisingly found most of these proteins are unable to be detected by those studies except BSK1 [53–55] and BES1 [51, 53, 54]. These results might be due to several reasons. First, the physicochemical properties of these proteins might be limited to detect their phosphorylation by current techniques. Second, BAK1 and BSK1 belong to kinases, which are low cellular abundant proteins compared with their substrates to shield theirs signals [57]. Third, previous studies showed that BR application could lead BAK1 phosphorylation within 30 s and reduces it after 120 s that might cause BAK1 not to be found in our study [58]. Finally, BRI1 and BKI1 are transmembrane proteins which are not easy to be extracted due to their hydrophobic character [59].

Several BR-signaling associated proteins such as GF14 PHI, SK 11, ATSK12, SK13, GSK1, MPK3, MPK6, BSL1, and BSL3 were detected by our phosphoproteomics experiments and PIN analysis. However, these proteins in our study did not reveal significant phosphorylation changes after BR treatment. The possible reason is that most of them might be inhibited or played as negative regulators in BR signalling [38]. Nevertheless, we still identified some BR-signaling related proteins, such as BSK3 [60] and BIL4 [49] which revealed differential phosphorylation expression after BR treatment. It indicates that our phosphoproteomics experiment can identify known BR signaling and capture potential novel BR signals.

Because the interactome of *Arabidopsis* is incomplete, several known BR-related proteins identified in our data were not incorporated into PIN. For example, relative of early flowering 6 (REF6) and beta-amylase 7 (BAM7) are two important regulators of BR responsive gene expression. REF6, a Jumonji domain–containing protein, interacts with BES1 to regulate target gene expression and coordinates BR response with other development processes [61]. BAM7, a beta-amylase-like protein, contains high similarity to the N-terminal DNA-binding domain of BZR1. Therefore, BAM7 protein can bind to the *cis*-regulatory element similar to BZR1 but has opposite effect to BZR1 on gene expression [62].

**Fig. 8:**
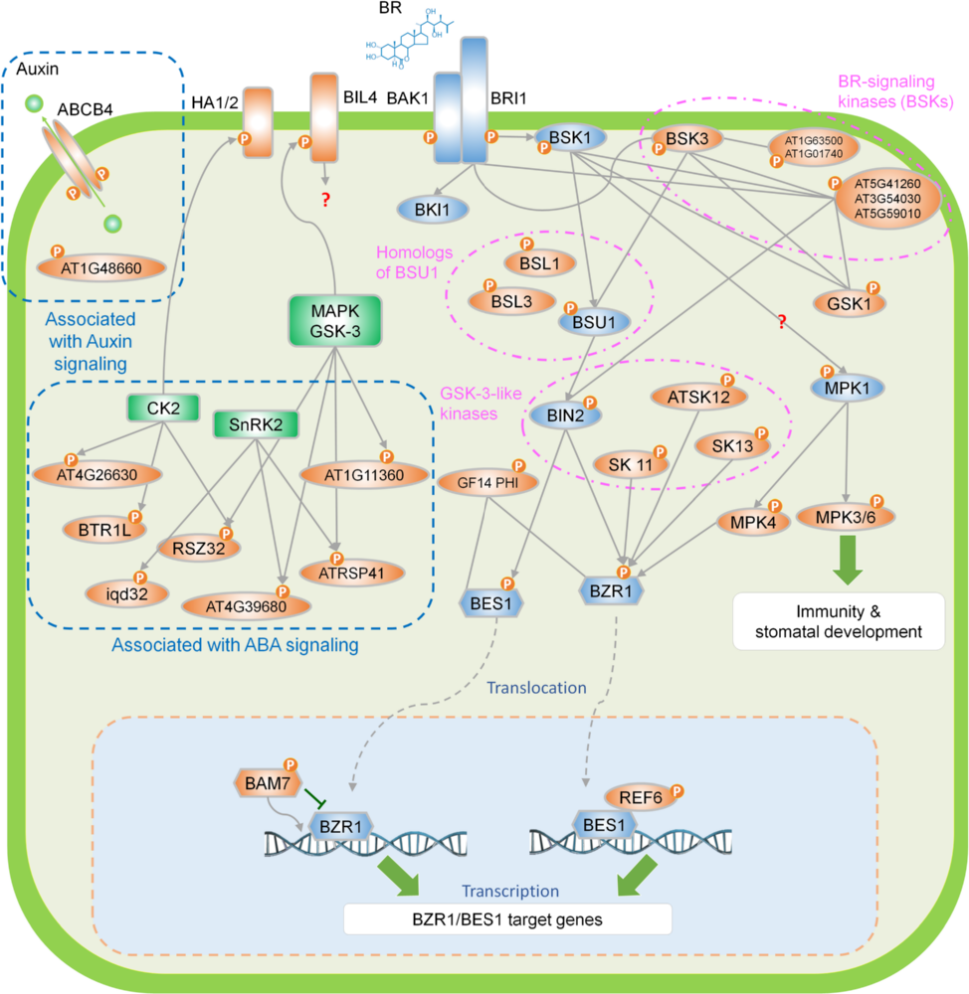
Illustration of known and hypothesized BR signaling pathway. Integration of identified phosphoproteins, proposed the kinases and its substrates network from this study, as well as known BR response signals from literature, the BR phosphosignaling network was constructed. The crosstalk between BR response, auxin and ABA signaling was displayed. The *orange*, *green* and *blue* nodes denote the identified phosphoproteins in this study, the kinase, and the BR-related proteins, respectively. The interaction or relationships between proteins are obtained from literature and our bioinformatics inference

Although only a quarter of phosphorylation sites were quantified at all five time points, our bioinformatics approaches help to reveal the dynamics of BR regulation. First, GO enrichment analysis showed the transition of biological processes after BR treatment. Then, we used *Arabidopsis* interactome and sequence content of phosphorylation site to connect and interpret these phosphoproteins and intended to unravel the complexity mechanisms of BR signaling (Fig. 8). In addition to BSK3, other homologs of BSK, including AT5G59010, AT3G54030, and AT5G41260, were also detected and had the consensus phosphorylation site in BSK3. Recent study demonstrates redundant biological functions for BSKs and suggests the existence of a regulatory link between BSKs and GSK3-like kinases [60]. The motif analysis results showed that the substrates of GSK3/MAPK were significantly regulated. Current research indicates that GSK3-like kinases play the crucial roles in BR signalling [6, 7] as well as many physiological processes were regulated by MAPK, such as stomatal development and plant immunity [63]. Therefore, the substrate of GSK3/MAPK might be considered as the downstream of BR signaling. We also compared the proteins with significantly phosphorylation changes to BR-responsive genes in transcription level and suggested that the phosphorylation difference on most of proteins observed in this study, especially at 5 and 30 min, might be directly affected by BR signaling, not indirectly due to transcriptional regulation of BR target genes.

The constructed time-dependent kinase-substrate interaction networks may also indicate the possible mechanism of crosstalk between BR and other hormone signaling (Fig. 8). BR might activate auxin signaling by positively phosphorylating on ABCB4 which regulates cellular auxin levels, so that we observed auxin-responsive GH3-like protein (AT1G48660) was in its active form [64]. CK2 and SnRK2 are important regulators in ABA signalling [45, 65], and we also observed their substrates were significantly regulated at 3 h of BR treatment. In previous studies, their substrates, including BTR1L, RSZ32, ATRSP41, AT4G39680, and AT4G26630, were shown to have an up-regulated level after ABA induction [51]. Interestingly, these proteins have a significantly phosphorylation down-regulation level at 30 min in our data. It implies that although the BR and ABA interact to regulate protein phosphorylations, the molecular mechanisms seems to be complex and required more information to be clarified.

In the end, the MS-based identified proteins with significantly phosphorylation changes might result from gene or protein expression changes, therefore we compared BR-responsive genes in phosphorylation and transcription levels and found low overlap between these gene set. This result can be used as an indirect evidence to demonstrate that observed BR-responsive phosphoproteins in our quantitative phosphoproteomics analysis are not affected by transcriptional regulations. Since this result might be due to the differences of experimental conditions, we pooled all BR-responsive genes under different conditions together to eliminate the effect. However, further experimental validations are required to clarify the novel BR-responsive phosphorylation events.

## Conclusions

This study provides a dynamic profile of phosphoproteome coupling bioinformatics to uncover the BR-regulated networks in *Arabidopsis*. Phosphoproteome analysis identified 1104 unique phosphopeptides from 739 unique phosphoproteins, as well as 1231 high-confidence phosphorylation sites, of which 239 were significantly regulated after treatment with BR. In initial BR responses, the known BR-related genes *BIL4* and *BSK3* with novel phosphorylation sites were identified. The motif enrichment on significantly regulated peptides reveals that BR signalling is regulated by kinases CK2, GSK3/MAPK, and SnRK2. Furthermore, these kinases play the important roles in BR interconnection with other plant hormone pathways, such as auxin and ABA. The phosphoproteins and phosphosites identified in our study provide a useful dataset for revealing signaling networks of BR regulation, and also expanded our knowledge of protein phosphorylation modification in plants as well as further deal to solve the plant growth problems.

## Methods

### Plant material and growth conditions

Plant System Biology Dark type (PSB-D) cell suspension cultures (*A. thaliana* ecotype Landsberg erecta) were maintained in 50 mL Murashige & Skoog medium (4.43 g/L; Duchefa Biochemie, Netherlands), 30 g/L sucrose, 0.5 mg/L naphthalene acetic acid, 0.05 mg/L kinetin; adjusted to pH 5.7 with 1 M KOH) at 27 °C under gentle agitation (130 rpm) in the dark. Cells were subcultured in fresh medium at a 1/10 dilution every seven days.

### BR treatment

For hormone starvation of PSB-D cells, cultured medium in 7-day-old cultures was replaced with fresh medium and maintained for 48 h, and then 24-epibrassinolide (a highly active BR; Sigma-Aldrich, USA) and DMSO (mock) were added to the medium. 24-epibrassinolide concentrations were 10 nM, 100 nM, 1 μM, and 5 μM in the gene expression assay, and 5 μM in the phosphoproteome analysis. Treatment times were chosen as 3 h in the gene expression assay, and 5 min, 30 min, 3 h, 6 h, and 12 h in the phosphoproteome analysis. Cells (0.2 g, wet) were immediately washed with 0.1 M phosphate buffer (pH 5.7) and frozen in liquid nitrogen in preparation for further experiments.

### Real-time PCR

Total RNA from plant cells was isolated with TRIzol Reagent (Invitrogen, USA) and cDNA was synthesized using a RevertAid H Minus reverse transcriptase kit (Fermentas, USA) following manufacturer’s instructions. Primer design is shown in Additional file 6. Gene expressions regulated by BR were detected using an iQ5 Real-time PCR detection system (Bio-Rad) with a KAPA SYBR® FAST One-Step qRT-PCR Bio-Rad iCycler kit (KAPA Biosystems, USA) following manufacturer’s instructions.

### Protein extraction and trypsin digestion

Proteins were extracted by 0.1 M Tris–HCl (pH 8.0) and 8 M urea with protein phosphatase inhibitor cocktails (Bioman, Taiwan) and protease inhibitors (BioShop, Canada). Protein concentrations were quantified using a Bio-Rad Protein Assay (Bio-Rad, Munich, Germany). The protein extracts of each sample was reduced with 10 mM dithiothreitol (Wako, Japan) for 45 min at room temperature, and then carbamidomethylated with 50 mM iodoacetamide (Sigma, USA) for 45 min at room temperature in darkness. Alkylated proteins were diluted four times with 50 mM triethylammonium bicarbonate (TEAB) and digested with endopeptidase Lys-C (1:100 w/w) (Wako) for 2 h. Subsequently, sequencing-grade modified trypsin (1:100 w/w) (Promega, Germany) was added for 16–18 h at room temperature. The digested peptides were acidified to a pH < 3 with trifluoroacetic acid (TFA). Acidified peptides were desalted using StageTips with SDB-XC Empore disc membranes (SDB-XC StageTip) (3 M, Germany) [66], and eluted in a buffer containing 0.1 % TFA and 80 % acetonitrile.

### Dimethylation labeling

All peptide concentrations were quantified using the BCA protein assay (Pierce Biotechnology, Inc., Rockford, IL, USA) as per the manufacturer’s instructions. 200 μg tryptic peptide of each sample were dried and re-dissolved in 0.1 M TEAB. Mock-treated samples were labeled with 4 % formaldehyde-*H*_*2*_. BR-treated samples were labeled with formaldehyde-*D*_*2*_. And then, 6 μL of freshly prepared 0.6 M sodium cyanoborohydride was immediately added to sample solution. The solution was mixed for 60 min at room temperature, and then 1 % ammonium hydroxide was added to stop the reaction. Formic acid was used to further stop the reaction and acidify the samples. Finally, the labeled samples were combined at a 1:1 ratio and desalted using SDB-XC StageTip.

### Phosphopeptide enrichment

The phosphopeptide was enriched by HAMMOC as previously described [19, 66–68]. Custom HAMMOC tips were prepared by packing TiO_2_ beads (10 μm, GL Sciences, Japan) into C8 StageTips. Prior to sample loading, HAMMOC tips were equilibrated with solution A containing 0.1 % TFA, 5 % acetonitrile, and 300 mg/mL lactic acid. All peptide concentrations were quantified using the BCA protein assay (Pierce Biotechnology, Inc., Rockford, IL, USA) as per the manufacturer’s instructions. About 400 μg desalted peptide mixture was mixed with an equal volume of solution A and loaded onto the HAMMOC tips (100 μg mixed peptides per tip). After successive washing with solution A and solution B (0.1 % TFA and 80 % acetonitrile), the resulting phosphopeptides were eluted by 0.5 and 5 % piperidine (Wako). The eluent was acidified with TFA, desalted with the SDB-XC StageTip, and vacuum-dried. The phosphopeptides were re-suspended in 0.5 % TFA and subjected to nanoLC-MS/MS analysis.

### NanoLC-MS/MS analysis

For mass spectrometry analysis, LC was performed on an Agilent 1100 series HPLC system (Agilent Technologies, USA) with a micro-T for flow splitting coupled to an LTQ-Orbitrap XL hybrid mass spectrometer (Thermo Electron, USA) equipped with a PicoView nanospray interface (New Objective, USA). Peptide mixtures were loaded onto a 75 μm × 250 mm fused silica capillary column packed in-house with C18 resin (5 μm, Nucleosil 120–5 C18; Macherey-Nagel, Germany), and were separated over 110 min using a linear gradient of 2–50 % solvent B (95 % acetonitrile with 0.1 % formic acid) and solvent A (0.1 % formic acid in water) at a flow rate of 300 nL/min. The LTQ-Orbitrap was operated in positive ion mode and mass spectra from full scans were acquired on the Orbitrap analyzer (m/z 350–1600) with resolution set to R = 30,000 (at m/z 400). The ten most intense peptide ions were selected from the MS scan and fragmented by collision-induced dissociation for MS/MS scan in the LTQ analyzer. All Orbitrap measurements were performed with the “lock mass” option to improve mass accuracy of precursor ions.

### Data analysis and database search

Raw spectrum files were processed for phosphopeptide identification and phosphosite quantification with MaxQuant software version 1.3.0.5 (http://maxquant.org/) [69]. Peptide identification was performed using Andromeda search engine [70] against the *Arabidopsis* TAIR10 database (http://www.arabidopsis.org/; version pep_20101214). Search criteria were trypsin specificity, fixed modification of carbamidomethyl, variable modifications of oxidationand phosphorylation, and two allowed missed cleavages. A minimum peptide length of seven amino acids was required. Precursor mass tolerance was set at 6 ppm, and fragment ion tolerance at 0.5 Da. A target-decoy search strategy was used in this study [71]. Protein, peptide, and site identification were accepted on the basis of posterior error probability with a false discovery rate (FDR) of 1 %. Precursors of already identified peptides were further searched using the “match between runs” option in MaxQuant, which matches precursor masses in a 2 min retention time window. The localization probability of all putative phosphorylation sites was determined using the MaxQuant post-translational modification score algorithm. The proteomics data have been deposited to the ProteomeXchange Consortium [72] via the PRIDE partner repository with the dataset identifier PXD001473. Proteins were counted separately if a peptide matched to multiple proteins in the database search. Identified phosphorylation sites were grouped into three classes based on the phosphorylation localization probability score: class I (*p* > 0.75), class II (0.5 < *p* ≤ 0.75), and class III (*p* ≤ 0.5) [73]. Class I sites were considered as confident assignments and used for all analyses in the study.

### Construction of protein-protein interaction network

We collected protein-protein interactions of Arabidopsis from BioGRID [35],AtPID [36], and PhosPhAt [37]. Additional, the known BR signaling components were collected from literature [3, 6, 7]. The interactions which connect the two identified phosphoproteins or link identified phosphoproteins to the known BR-related proteins were assembled into a network. The constructed network was visualized by Cytoscape [74].

### Gene ontology enrichment analyses

The Gene Ontology (GO) annotation for all *Arabidopsis* proteins was obtained from TAIR [75]. The one-sided Fisher’s exact tests were performed to identify the over-representative GO terms. *P*-values returned by one-sided Fisher’s exact tests were adjusted via the Benjamini–Hochberg method to control false discovery rate (FDR). The enrichment analysis for biological process (BP) and cellular component (CC) ontologies were done separately for each of time points.

For further hierarchical clustering based on BP GO terms, we first collated all the GO terms obtained after the enrichment, and the filtered for those GO terms which were at least enriched in one the of time points with adjusted *P*-value < 0.05. This filtered *P*-value matrix was transformed by the function *x* = −log_10_ (*P*-value). For each GO term, the mean (*μ*) and standard deviation (*ρ*) of these *x* values were calculated, and then *x* values were z-transformed by following formula:

$$ z=\frac{x-\mu }{\rho } $$

These z scores were then clustered by one-way hierarchical clustering by Pearson correlation coefficient and average linkage clustering in GAP (Generalized Association Plots) [76].

### Phosphorylation motif analysis

All confident phosphopeptide sequences were submitted to Motif-X (v1.2) [77] for the identification of over-represented motifs. Sequences were centered on each phosphorylation site and extended to 15 amino acids (±7 residues). Where the phosphorylation site was close to the N/C-terminal of the protein, the sequence was filled with up to 15 amino acids with the required number of “X” which denotes any amino acid. The significance threshold was set to *P* < 10^−6^ and the occurrence threshold, the minimum number of times you wish each of your extracted motifs to occur in the data set, was set to 30 for Ser and 20 for Thr and Tyr. The *Arabidopsis* International Protein Index (IPI) database provided by Motif-X was used as a background. Over-represented motifs were associated with specific kinases by searching manually the literature and databases, including PhosPhAt (the *Arabidopsis* phosphorylation site database) [37], HPRD (Human Protein Reference Database) [42], and PhosphoNetworks [43]. A motif-kinase association was constructed if the motif was identical to the recognizing pattern of a given kinase reported by literature or described in a database.

To reveal the dynamics of kinase regulations, we assessed the situation of each enriched motif at each time point. For a motif *m* at the time point *t*, one side-Fisher’s exact test was performed using the 2 × 2 contingency table that include the following numbers: *k, K-k, n, N-n* where *k* denotes the number of peptides which intensity ratios at *t* pass a threshold and contain the motif *m*, *K* is the total number of peptides which intensity ratios at *t* pass a threshold (i.e. |log_2_ (fold-change)| ≧ 1), *n* denotes the number of peptides which are identified at *t* and contain motif *m, and N* is the total number of peptides identified at *t.* We analyzed separately the up- and down-regulated peptides.

### Comparison between differential phosphorylation proteins and expression genes of BR-responsive

The BR-responsive genes derived from genome-wide transcriptional experiments were obtained from Wang and colleagues [6]. The overlap between differential phosphorylation protein set and differential expression gene set was assessed by Fisher’s exact test.

## Additional files

Additional file 1:
**The list of all quantified phosphorylation sites.**
Additional file 2:
**The distribution of phosphorylation sites with missing quantification.**
Additional file 3:
**Biological process ontology enrichment analysis at each time point.**
Additional file 4:
**Arabidopsis consensus motifs correspond to human kinases).**
Additional file 5:
**Identified phosphoproteins also involving in expression regulation by BR induction.**
Additional file 6:
**Primer list in this study.**

## Abbreviations

ABA: Abscisic acid
ABCB4: ATP-binding cassette sub-family B member 4
AtPID: Arabidopsis thaliana Protein Interactome Database
ATSK12: Shaggy-related kinase 12
BAK1: Bri1-associated receptor kinase 1
BAM7: Beta-amylase 7
BES1: Bri1-ems-suppressor 1
BIL4: Brz-insensitive-long hypocotyls 4
BIN2: Br-insensitive 2
BKI1: Bri1 kinase inhibitor 1
BP: Biological process
BR: Brassinosteroid
BRI1: BR-insensitive 1
BRU6: Indole-3-acetic acid amidosynthetase
BSK: BR-signaling kinase
BSU1: Bri1 suppressor1
BZR1: Brassinazole-resistant 1
CC: Cellular component
CK: Mock
CSNK1G3: Casein kinase 1, gamma 3
DYRK4: Dual-specificity tyrosine-phosphorylation regulated kinase 4
FDR: False discovery rate
FLS2: Flagellin-sensing 2
GAP: Generalized Association Plots
GF14 PHI: 14-3-3-like protein G-box factor 14 phi
GO: Gene Ontology
GSK1: Shaggy-like protein kinase 1
GSK3: Glycogen synthases kinase 3
HAs: H(+)-ATPases
HAMMOC: Aliphatic hydroxy acid-modified metal oxide chromatography
HPRD: Human Protein Reference Database
IMAC: Immobilized metal-affinity chromatography
Iqd32: IQ-domain 32
LAST1: Large tumor suppressor kinase 1
MAPK: Mitogen activated protein kinase
MKK4/5/7/9: Mitogen-activated protein kinase kinases 4, 5, 7 and 9
MPK3/6: Mitogen-activated protein kinase 3 and 6
NanoLC-MS/MS: Nanoliquid chromatography-tandem mass spectrometry
NEK2: NIMA-related kinase 2
PDIK1L: PDLIM1 interacting kinase 1 like
PhosPhAt: Arabidopsis phosphorylation site database
PIN: Protein-protein interaction network
PRPF4B: Pre-mRNA processing factor 4B
PSB-D: Plant System Biology-Dark type culture
REF6: Relative of early flowering 6
RLCK: Receptor-like cytoplasmic kinases
SAUR-AC1: Small auxin up RNA
SCX: Strong cation exchange
SK 11: Shaggy-related kinase 11
SK13: Shaggy-related kinase 13
SnRK2: SNF1-related kinase II
TAIR: The Arabidopsis Information Resource
TEAB: Triethylammonium bicarbonate
TFA: Trifluoroacetic acid
TiO2: Titanium dioxide
YODA: Mitogen-activated protein kinase kinase kinase YODA
