# Supplementary material for: Integrating Phosphoproteomics and Bioinformatics to Study Brassinosteroid-Regulated Phosphorylation Dynamics in Arabidopsis
Source: BMC Genomics. 2015 Jul 18;16(1):533. doi: 10.1186/s12864-015-1753-4 (PMC4506601; doi:10.1186/s12864-015-1753-4)
Supplement: Additional file 2: — The distribution of phosphorylation sites with missing quantification. [file 12864_2015_1753_MOESM2_ESM.docx]

**Additional file 2. The distribution of phosphorylation sites with missing quantification.** The missing time point means a phosphosites at a given time point can’t be quantified by MaxQuant analysis.
