# Supplementary material for: Integrating Phosphoproteomics and Bioinformatics to Study Brassinosteroid-Regulated Phosphorylation Dynamics in Arabidopsis
Source: BMC Genomics. 2015 Jul 18;16(1):533. doi: 10.1186/s12864-015-1753-4 (PMC4506601; doi:10.1186/s12864-015-1753-4)
Supplement: Additional file 4: — Arabidopsis consensus motifs correspond to human kinases). [file 12864_2015_1753_MOESM4_ESM.docx]

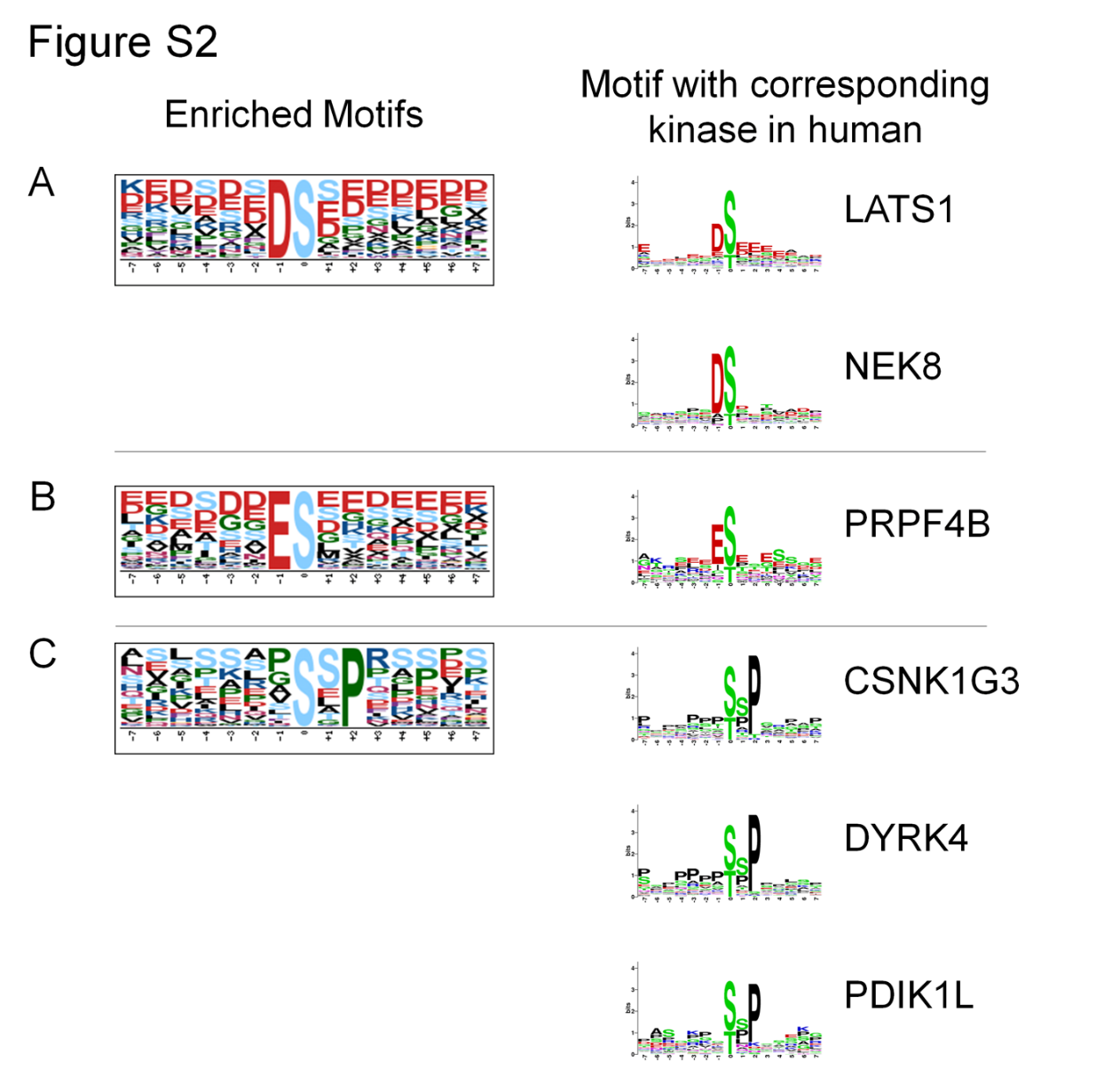


**Additional file 4. Arabidopsis consensus motifs correspond to human kinases**

Motif (A) DpS (B) EpS (C) pSXDXE were recognized from human kinases. The left motif logos were generated from Motif-X based on all identified phosphopeptides and the right motif logos were obtained from PhosphoNetworks (http://phosphonetworks.org/).
